# Basal pancreatic secretion in a comparative aspect in poultry and rodents

**DOI:** 10.3389/fphys.2024.1340130

**Published:** 2024-03-15

**Authors:** Irina V. Kuzmina, Svetlana M. Tolpygo, Alexander V. Kotov, Batogab B. Shoibonov, Tatyana S. Zamolodchikova

**Affiliations:** Laboratory Physiology of Motivations, Federal State Budget Scientific Institution “P. K. Anokhin Research Institute of Normal Physiology”, Moscow, Russia

**Keywords:** pancreas, poultry, rodents, basal secretion, trypsin

## Introduction

The pancreas is one of the central organs of the gastrointestinal tract, playing an active role in the regulation of the digestive system (Kuznetsov and Smelysheva, 2006). Pancreatic secretion contains enzymes that hydrolyze substrates of protein, lipid, and carbohydrate nature. It is known that the pancreatic enzyme trypsin, which belongs to the class of serine proteases, initiates the initial stage of protein hydrolysis in the intestine in most animal species. At the same time, there is literature evidence that trypsin has the same effect as pancreatic juice, and its inhibitor enhances pancreatic secretion when the pancreatic juice returns to the duodenum (Korotko, 2016). It can be hypothesized that pancreatic secretion undergoes negative feedback inhibition, which is activated by duodenal trypsin. Previously, several authors have concluded that basal pancreatic secretion in humans and rats is controlled in a negative feedback manner by intestinal pancreatic juice or tryptic activity (Korotko, 2010). Therefore, the question as to whether feeding and secretion patterns are not separate and independent phenomena are related to interdigestive activity but represent accompanying modifications arises. In this relation, in this paper, we want to examine, in a comparative aspect, how basal pancreatic secretion changes in animals with different types of nutrition (laying hens, broiler chickens, laboratory rats, and rabbits). This will allow in further expanding the ideas about the nature of participation of digestive enzymes in the regulation of physiological functions of the organism.

## Opinion

### Basal pancreatic secretion in poultry

The pancreas of birds secretes a relatively much larger amount of juice compared to mammals (Vertiprakhov and Fomenko, 2016). It is an organ of synthesis of digestive enzymes, the role of which in the processes of adaptation of digestive organs to the peculiarities of the chemical composition of food is of great importance. The work of the pancreas is directly affected by the type of nutrition and peculiarities of food specialization of birds (Shcheglov, 2011). In birds, the pancreas is sufficiently developed and the pancreatic juice is secreted in it constantly. Adult chickens secrete 25 mL of pancreatic juice, which is much more than in other animal types. The same enzymes have been found in the pancreatic juice of poultry as in mammals. It should be noted that pancreatic secretion in birds is insufficiently studied. Thus, the reasons for the secretion that continues during starvation, when chyme does not enter the intestine, have not been elucidated to date (Ayurzanaeva et al., 2017). It is known from the literature that the extrinsic secretory function of the pancreas of chickens is clearly adapted to the quality of the ingested feed, mainly due to changes in the enzymatic activity. The most perfect type of enzyme adaptations of the pancreas is apparently a change in the amount of enzymes necessary for digestion of incoming food by changing their concentration in the juice (Fisinin et al., 2018a). It is in this aspect that the processes of specific enzyme adaptation are manifested. If the organism cannot create a strictly defined concentration of enzyme in the juice, this process involves a less economical and less specific mechanism—an increase in the amount of secreted juice (Kuznetsov and Smelysheva, 2004). The amount of secreted enzymes, in this case, can change less specifically and, not so strictly, correspond to the quality of the feed consumed (Vertiprakhov and Svitkin, 2017). When analyzing the basal activity of digestive enzymes, including trypsin, it is shown that before feed intake and the first 30 min after its intake, the activity of enzymes practically does not change; however, there is an increase in the activity of enzymes due to the complex reflex phase of regulation of pancreatic secretion (Fisinin et al., 2018b). It was revealed that the juice starts to be secreted a few minutes after feeding and reaches its maximum value in 30 min, and then follows a decrease and a new enzymatic rise associated with the neurochemical phase of regulation, when the hormones secretin and pancreozymine start to be secreted in the duodenum under the action of the feed coming from the stomach (Rybakov, 2021). The basal secretion can be different in poultry, using different ingredient feeds. Proteolytic enzymes, including trypsin, can reach their peak activity at 120 min after feed intake. However, the very process of adaptation of proteolytic enzyme activity to different types of feed will take 3–5 days (Somova, 2012; Vertiprakhov et al., 2016). Several authors who measured the “basal exocrine secretionˮ of the pancreas in chickens have found inter- and intra-individual differences in the rate of secretion, which is cyclic and related to the motor function of the intestine (Salido et al., 1984; Vertiprakhov and Ovchinnikova, 2022).

### Basal pancreatic secretion in rodents

The mammalian pancreas is a gland of mixed type of secretion. It morphologically consists of two parts with different functions—exocrine and endocrine (Barteneva et al., 2022). In addition to birds, in rodents (rabbits and rats), under prolonged maintenance of a certain dietary intake, the secretory process in the pancreas undergoes adaptive changes, such as the pronounced basal secretion of electrolytes by the pancreas and its weak response to secretin (Aksyonova et al., 2021). The basal secretion of pancreatic enzymes in rabbits for 3 hours at 5-min intervals and stimulated secretion after the administration of cholecystokinin and methacholine chloride were studied (Adelson et al., 1995). It has been shown that there are differences in the percentage of each enzyme studied (amylase, lipase, chymotrypsinogen, and trypsinogen) to the total number of secreted enzymes or their ratios to each other, both under conditions of basal secretion and under hormonal (cholecystokinin) and neurotransmitter (cholinergic) stimulation. Changes in enzyme percentages and proportions were in complete agreement with changes in intra-pancreatic secretory enzyme sources. The degree of correlation between enzymes during secretion depended on the type of enzyme and on the stimulating factor. The heterogeneity of pancreatic protein sources has been suggested to involve the existence of many types of receptors on the acinar cell surface that facilitate the integration of different kinds of neutral and hormonal signals (Mozheiko, 2017).

In the studies on rats with pancreatic, biliary, duodenal, and jugular fistulas allowing separate drainage of bile and pure pancreatic juice, it was revealed that in spite of animal starvation, during 10 h before the beginning of the experiment and during the whole 6-h experimental period, pancreatic juice was continuously secreted. This made it possible to collect pancreatic juice every 15 min throughout the experiment to determine the activity of digestive enzymes in it. The results obtained indicate that under fasting conditions, somatostatin and atropine can neutralize the basal output of pancreatic enzymes, which accounts for the constitutive type of secretion characterized as parallel secretion of digestive enzymes. In addition, it is suggested that under conditions of basal secretion, acetylcholine and cholecystokinin entering pancreatic acinar cells may contribute to the dissociation of pancreatic secretion of individual digestive enzymes originating from heterogeneous secretory granules (Toriumi et al., 1994). K.O. Miyasaka, on a similar experimental model on rats, stated that the regulation of the intestinal lumen is carried out by proteases on the principle of negative feedback. In this case, the increase in pancreatic secretion does not occur until the activity of proteases in the intestine decreases by more than 90% (Miyasaka and Gary, 1984). These results suggest that inhibition of pancreatic secretion by proteases contained in pancreatic juice is much more effective than inhibition by proteases (e.g., trypsin) administered alone. It can be concluded that exocrine pancreatic secretion is mainly regulated by a feedback mechanism, through trypsin activity in the intestinal contents.

### Basal trypsin secretion in birds and mammals

A number of studies have demonstrated a reversible inhibition of pancreatic enzyme secretion by pancreatic proteases in the duodenum in animals. Several authors have shown that secretion of pancreatic bile juice from the proximal intestine causes a marked increase in pancreatic enzyme secretion. Trypsin, chymotrypsin, or pancreatic bile juice injected into the duodenum suppressed pancreatic enzyme secretion. Although a similar feedback control system has been found in chickens and pigs, no such regulatory control exists in the dog (Owyang et al., 1986). Basal secretion of trypsin is known to decrease during starvation (Abou-Assi et al., 2001). In studies on rats, it was found that there is a decrease in serum trypsin activity during starvation (Kuzmina et al., 2023). Furthermore, basal secretion of trypsin was markedly reduced in diabetic rats compared to that in normal rats (Shimizu et al., 2000). Thus, it can be assumed that all common forms of enteral nutrition stimulate pancreatic trypsin synthesis and secretion, whereas the starvation state decreases it. The manifestation of various diseases related to pancreatic function may also affect basal secretion of trypsin, thereby reducing the activity of this enzyme.

## Discussion

The processes of neurohumoral regulation of the gastrointestinal tract are complex and ensure the maintenance of adequate secretion of pancreatic enzymes, which are necessary for adequate digestion of consumed food (Rodger, 2018). The pancreas is an integrated, well-adjusted organ, all components of which are closely interconnected and probably interchangeable. The morphological substrate of this unified endocrine–exocrine–ductal axis is the intrapancreatic “portal” capillary system, enzyme–hormonal system, and ultrastructural features of acino-ostrophic cells, which ensures maximum efficiency of this organ. It is well-known that pancreatic secretion continues at a basal rate during fasting, with fluctuations coinciding with migratory motor complexes. Basal pancreatic secretion is maintained not simply by the slow release of zymogen stores but also by the fact that enzymes continue to be synthesized to replace secretions and maintain the zymogen pool at a reduced size.
